# Left Atrial Appendages from Adult Hearts Contain a Reservoir of Diverse Cardiac Progenitor Cells

**DOI:** 10.1371/journal.pone.0059228

**Published:** 2013-03-12

**Authors:** Jussi V. Leinonen, Avishag K. Emanuelov, Yardanna Platt, Yaron Helman, Yael Feinberg, Chaim Lotan, Ronen Beeri

**Affiliations:** Cardiovascular Research Center, Heart Institute, Hadassah Hebrew University Medical Center, Jerusalem, Israel; Tokai University, Japan

## Abstract

**Aims:**

There is strong evidence supporting the claim that endogenous cardiac progenitor cells (CPCs) are key players in cardiac regeneration, but the anatomic source and phenotype of the master cardiac progenitors remains uncertain. Our aim was to investigate the different cardiac stem cell populations in the left atrial appendage (LAA) and their fates.

**Methods and Results:**

We investigated the CPC content and profile of adult murine LAAs using immunohistochemistry and flow cytometry. We demonstrate that the LAA contains a large number of CPCs relative to other areas of the heart, representing over 20% of the total cell number. We grew two distinct CPC populations from the LAA by varying the degree of proteolysis. These differed by their histological location, surface marker profiles and growth dynamics. Specifically, CD45^pos^ cells grew with milder proteolysis, while CD45^neg^ cells grew mainly with more intense proteolysis. Both cell types could be induced to differentiate into cells with cardiomyocyte markers and organelles, albeit by different protocols. Many CD45^pos^ cells expressed CD45 initially and rapidly lost its expression while differentiating.

**Conclusions:**

Our results demonstrate that the left atrial appendage plays a role as a reservoir of multiple types of progenitor cells in murine adult hearts. Two different types of CPCs were isolated, differing in their epicardial-myocardial localization. Considering studies demonstrating layer-specific origins of different cardiac progenitor cells, our findings may shed light on possible pathways to study and utilize the diversity of endogenous progenitor cells in the adult heart.

## Introduction

During the last decade there has been evidence supporting the regenerative capability of the adult heart, but the mechanism is still debated. There is strong evidence supporting the claim that endogenous cardiac progenitor cells (CPCs) are key players in cardiac regeneration, but the anatomic source and transcriptional phenotype of the master cardiac progenitors remains uncertain [1]. CPCs are a heterogenic group and are thought to be concentrated in specific areas of the heart, e.g. atria or epicardium [2], [3].Non-myocyte cells are the predominant cell population of the heart in number: cardiomyocytes are estimated to constitute 75% of the normal myocardial tissue volume in murine hearts, but only 30–55% of the total cell number [4]. This enables great variability in the cellular composition between different cardiac structures.

Embryonic cardiogenesis has been lately shown to be much more diverse than previously thought [5]. The heart contains multiple complex structures, which originate from distinct cell types. Mesodermal-derived first and secondary heart field cells contribute most of the structures, but a number of cells originate from the cardiac neural crest. The pro-epicardium, on the other hand, gives rise to the adult epicardium, but possibly also contributes to the cardiac chamber formation [6]. Epicardial cells retain, in adulthood, the potential to activate embryonic transcription factors in response to cardiac injury [7].

**Figure 1:**
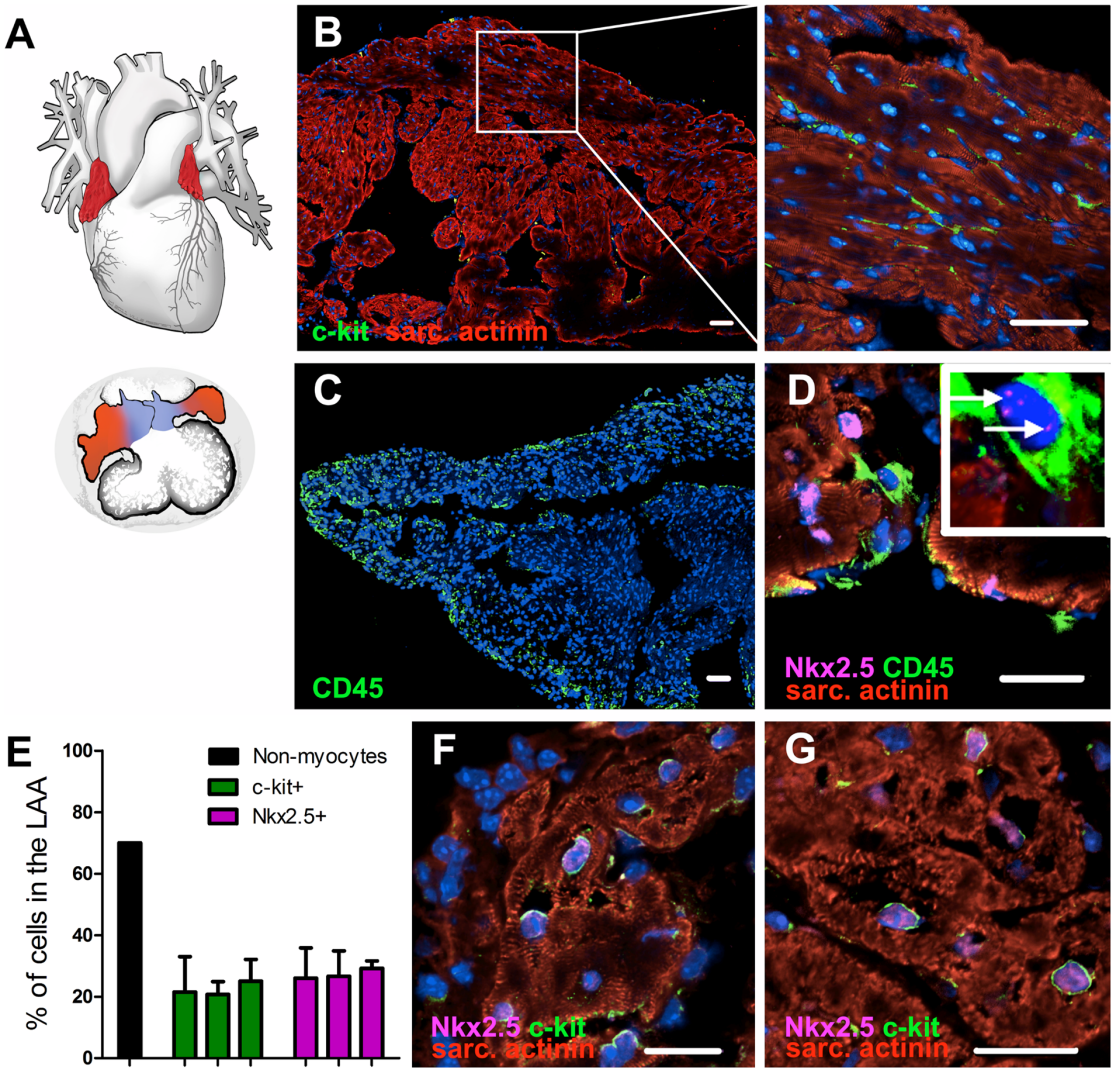
Anatomy and histology of the left atrial appendage. (A) Location and size of the atrial appendages (red) in the adult heart (top) and in the end stage of heart looping (bottom). Atria (blue) are still small and poorly developed compared to the appendages. (B) A vast population of c-kit^pos^ cells between the cardiomyocytes is found in the LAA of a 12w old mouse (bar  = 50 µm). (C and D) CD45^pos^ cells are concentrated in the near vicinity of the epicardium of the LAA with a weaker expression of Nkx2.5 (arrows in the magnified CD45^pos^ cell), bar  = 50 µm. (E) Percentage of cells expressing c-kit or Nkx2.5 was compared to the total number of nuclei in the LAA using computer software (Cellprofiler 2.0). Estimated percentage of non-myocyte cells in the whole murine heart, according to the literature [4], is added as a reference. Results obtained from three LAAs are shown in their representative graphs using five threshold levels (mean +SD). (F and G) Evidence of co-expression of c-kit and Nkx2.5 in the CPCs (bar  = 50 µm). Abbreviation: sarc. actinin  =  sarcomeric α-actinin. DAPI  = blue.

The atrial appendages (Figure 1A) have several unique features. They have a different embryonic origin compared to the atria, as the formation of the two appendages differentiates the morphologically right and left sides of the primary atrium [8]. At a later stage of the development the sinus venosus sprouts the pulmonary vein, which will eventually form the atria and the inter-atrial septum [9]. In addition, the atrial appendages have a distinctive anatomy. While the atria are smooth-walled, the appendages contain numerous trabeculae (pectinate muscles), resembling the ventricles. Interestingly, the epicardium on the surface of the atrial appendages is significantly thicker than over the ventricles [10]. In addition, the LAA lies in close epicardial contact to the left ventricle within the confines of the pericardium. The atrial appendages also function as storage for atrial natriuretic factor (ANF). In normal hearts, 30% of the ANF is contained in the LAA [10]. High concentrations of ANF correspond to high activities of the NPPA -gene, which is linked to early development of the heart and to fetal gene reprogramming during heart failure [11]. Research about human CPCs is often performed on right atrial appendage (RAA) tissue, which is removed during open-heart surgeries [12].

The estimated CPC density in an adult murine heart is one cell every 10^4^ myocytes [13]. We hypothesized that the left atrial appendage contains CPCs in greater concentration than the published estimate. We chose to investigate the left atrial appendages of mice, because of the easy and accurate removability of the LAAs without contamination from atrial tissue, which is more difficult for the RAAs. Two c-kit expressing CPC populations has been found from the adult heart. CPC population co-expressing CD45 and c-kit, was found in the failing human [14] and feline hearts [15], in contrast to more extensively studied CPC population co-expressing Sca-1 and c-kit [13]. Resident stem cell population expressing CD45 has been found in skeletal muscle as well [16]. The effect of enzymatic digestion in producing specific progenitor cell types is debated [17], [18]. There is evidence that enzymatic digestion potency influences the specific myocardial layer origin [6]. Thus we hypothesized that a vast numbers of cardiac progenitor could be grown from the murine LAA; that different populations of CPCs could be differentially grown from the epicardial or deeper layers of the LAA by modulating its enzymatic digestion; and that these populations will differ phenotypically.

## Methods

### Ethics

The Hebrew University Animal Ethics Committee approved all animal studies, and experiments conformed to the Guide for the Care and Use of Laboratory Animals published by the United States National Institutes of Health.

### Tissue extraction & handling

Adult (8–12 weeks old) C57BL/6 mice were deeply anesthetized with isoflurane and LAAs were removed. The explant pieces were then digested three times (5 min) with trypsin & collagenase D. Strong enzyme digestion was performed with trypsin 0.25% and collagenase D 0.1% and weak enzyme digestion with trypsin 0.05% and collagenase D 0.1%. After the three digestions the explants were placed on 24 –well plates (tissue culture treated) in culture medium (CEM: Isocove’s modified Dulbecco’s Medium with 25 mM HEPES, supplemented with 10% FBS (fetal bovine serum), 1% L-glutamine, 1% penicillin-streptomycin & 0.1 mM 2 -mercaptoethanol) Fast-growing explants were passaged before three weeks and slow-growing explants before five weeks of culture. The cells used for differentiation studies were supplemented with dexamethasone (25 nM, 48 h) or 5-azacytidine (0.1 µM, 7 days), which were added one week after passage. Fresh atrial appendage tissue was dissociated with collagenase D (0.1%) for 40 min in 37°C, and the cell suspension was passed through a 40 µm filter for flow cytometry analysis.

### Immunocytochemistry and immunohistochemistry

Immunocytochemistry was performed in Ibidi μ-slide 8-well plates (IbiTreat or Poly-L coating). Fixation was performed with formaldehyde 4% and permeabilization with triton 0.2% or with absolute methanol (2 min, −20°C). Blocking was performed with 1% BSA in PBS. Primary antibodies were incubated overnight in 4°C or 1–2 h in room temperature. Secondary antibodies were incubated for 40 min in room temperature. SlowFade Gold with DAPI (Invitrogen) was used as the mounting medium. Immunohistochemistry was performed on snap-frozen LAAs cut to 10 μm thickness. The samples were fixed with acetone (5 min, −20°C) and blocked with Casblock (10 min, Invitrogen). Antibody incubation times were same as described above. In multiple staining protocols each primary antibody was individually incubated overnight in 4°C, followed by the appropriate secondary antibody to achieve less cross-reactions between antibodies. LSM 710 confocal microscope was used (Carl Zeiss) and the pictures were processed with Zen 2009 -software.

Primary antibodies used were: c-kit (Santa Cruz), Gata-4 (Santa Cruz), Nkx2.5 (Santa Cruz), Cardiac myosin heavy chain (Abcam), Sarcomeric α-actinin (Sigma), TnI (Santa Cruz), ANF (Santa Cruz), Connexin 43 (Santa Cruz), N-Cadherin (Santa Cruz), Ki-67 (Santa Cruz), Sca-1 (BD Bioscience, PE conjugated), CD45 (BD Bioscience, FITC conjugated), CD34 (BD Bioscience, PE conjugated).

### Flow cytometry

Flow cytometry was performed with BD LSR II (BD Bioscience) or BD FACSAria III (BD Bioscience). The cells were first detached with non-enzymatic Cell Dissociation Solution (Sigma) (30 min, 37°C) and centrifuged (400 g, 5 min). Cell amount was adjusted using a hemacytometer to the test tubes. Wash buffer (PBS + 1% BSA + 0.02% Sodium Azide) was added to each test tube and Anti-Mouse CD16/CD32 (eBioscience) for 10 min. Appropriate conjugated antibodies: c-kit (PE-Cy7, BD Bioscience), CD45 (FITC, BD Bioscience), CD45 (Pe-Cy7, eBioscience) Sca-1 (PE, BD Bioscience), CD31 (APC, BD Bioscience) & isotype controls were incubated in for 30 min.

### Tissue analysis

Programmed counting was performed with Cell Profiler 2.0 (GNU General public license version 2) using adjusted intensity and threshold variables. Original intensity value was adjusted to resemble the staining pattern most accurately and increased threshold levels resulted in an obviously lower number of cells counted that would be assumed. Calculations were performed from three LAAs (c-kit: 2497 nuclei & Nkx2.5: 1908 nuclei). Manual counting, using double staining with c-kit and Nkx2.5, was performed on three LAAs (284 nuclei) as well.

### Comparison of phase-bright cardiosphere-like cell growth from the tissue explants

Comparison of phase-bright cardiosphere-like cell growth from the LAA, RAA and ventricular apex explants (12-16w old mice) was done after ∼4 weeks of culture. Cells were grown using three different tissue digestion methods (n = 6 , two experiments were prepared using each method): high (0.25% trypsin & 0.1% collagenase D), low (0.05% trypsin & 0.1% collagenase D) or no digestion. Tissues were prepared from 2 mice/experiment. Weight of the LAA, RAA and apex tissue was adjusted near equal before cutting and all of the tissue pieces were cut to three or four explants.

### RNA extraction & RT-PCR

Total RNA was extracted with High Pure RNA isolation kit (Roche). cDNA was created via RT-PCR using oligo(dT). PCR reaction was polymerized using Taq PCR Master Mix (Qiagen). The primers that were used for gene amplification: mMCP-6 (Tpsb2), 5′ primer CTG GCT AGT CTG GTG TAC TC; 3′ primer CAG GGC CAC TTA CTC TCA GAA, Tm 60°C; MITF, exon 1a, 5′-AAGTCGGGGAGGAGTTTCAT-3′; exon 2, 5′-TGTGGTACTTGGTGGGGTTT-3′; The primers used as reversed were taken from exon 5 (5′-ACCATCAGCAACTCCTGTCC-3′). Due to the complexity of cycling conditions for MITF amplification, the information is provided individually if needed.

### Statistical methods

Explant growth data was analyzed by a one-way ANOVA. Bonferroni’s corrected t-test was used to compare the three groups. Surface marker expressions in FACS analysis were compared using two-tailed unpaired t-test, P-values <0.05 were considered significant. Data is presented as mean +SD.

## Results

### Histology of the murine left atrial appendage

Immunostaining revealed a vast number of small c-kit^pos^ (CD117) cells [19] located in the myocardium between the cardiomyocytes, and as well in the epicardium and endocardium (Figure 1B). Staining for CD45 demonstrated that the epicardium, and to a lesser extent the endocardium, contains a population of CD45^pos^ cells (Figure 1C, D). Only a small proportion of CD45^pos^ cells were found deep inside the myocardium.

**Figure 2:**
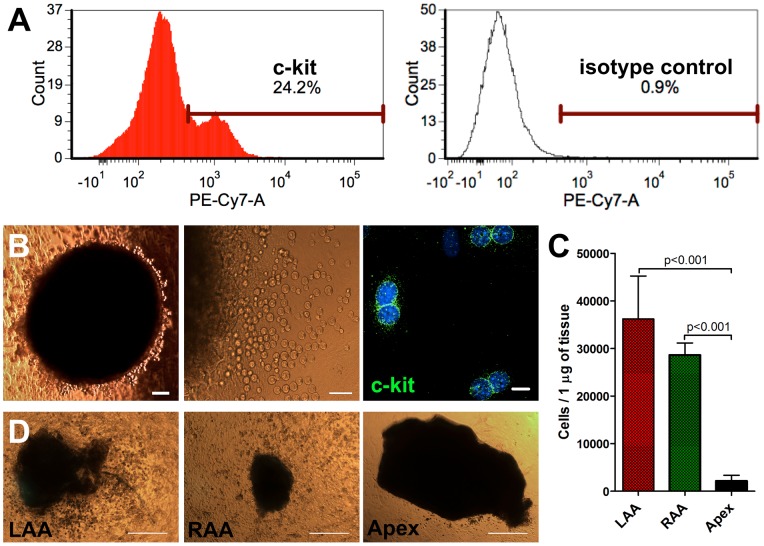
Estimated number of c-kit^pos^ CPCs in the atrial appendages. (A) Whole tissue dissociation of the both atrial appendages to single cell suspension from three adult mice showed a 24% cell population expressing c-kit, when analyzed using flow cytometry. (B) LAA explant (black) shown with increasing magnifications during an active growth phase of c-kit^pos^, phase-bright cells. (bars from left to right: 200µm, 100µm and 10µm). DAPI  =  blue. (C) Comparison of phase-bright cardiosphere-like cell growth from the LAA, RAA and ventricular apex explants after ∼4weeks of culture. At this time point ∼40–80% of the cells derived from LAAs and RAAs remained c-kit^pos^ (data not shown). Data is presented as mean+SD, n = 6. (D) LAA and RAA -explants produced significant quantities of cells. In contrast, apex-derived explants produced only a minimal number of cells (bar  = 500 µm).

We compared the number of c-kit^pos^ cells related to the total amount of nuclei (Figure 1E). In samples consisting of a near complete transverse cut of the LAA we discovered that the LAA contains a vast population of c-kit^pos^ cells, accounting for over 10% of the cells. We also found that Nkx2.5 [20], an early marker of cardiomyocyte progenitor cells, was strongly expressed in the LAA, accounting for over 20% of the cells (Figure S1A). Using a multiple-staining protocol we revealed that ∼77% of the cells that expressed Nkx2.5, expressed c-kit as well (Figure 1F,G; Figure S1B,C). CD45^pos^ cells were mainly located in the epicardium, which contained a portion of the c-kit^pos^ cell population (Figure S1D). Flow cytometry analysis, after a tissue dissociation of freshly dissected atrial appendages, demonstrated a high concentration of c-kit^pos^ CD45^neg^ CPCs as well, when compared to the number (one cell every 10^4^ myocytes) found in the literature [13] (Figure 2A). Thus, the LAA contains a vast number of c-kit^pos^ CPCs, which are mostly Nkx2.5^pos^.

### Growth of CPCs from the atrial appendage tissue explants

The LAAs of adult mice are small (∼3 µg) limiting the methods available to separate cells for *in vitro* studies. We used a previously published method [21]. to grow cardiosphere-like cells from tissue explants. We noticed that small pieces of LAA tissue produced high numbers of c-kit^pos^ cardiosphere-like cells (Figure 2B; Figure S2) without need of tissue priming of the CPCs with Thymosin beta4 treatment as published previously [22]. There are reported differences in CPC abundance in different areas of the heart^.^ We compared the amount of cells acquired from the atrial appendages and the ventricular apex, and found that the atrial appendages grew a similar amount of cells, but that the growth potential of the ventricular apex was minimal in comparison (Figure 2C, D; Figure S2).

Previous studies found that c-kit^pos^ CPCs grown from adult ventricular or atrial tissue started to sprout after 21 days [21], while a different type of CPC began sprouting from primed epicardium explants after only 6 days [2]. Likewise our explants demonstrated two different timing patterns, mainly related to the different concentration of proteolytic enzymes used. Most of the explants processed with a higher enzymatic concentration (0.25% trypsin & 0.1% collagenase D) started to sprout cells after 17–21 days (Type A), while most explants processed with a milder enzymatic concentration (0.05% trypsin & 0.1% collagenase D), or no proteolysis at all, started to sprout cells after 7–10 days (Type B). Thus the different dynamics of the CPC populations supported our theory that different method of proteolysis yields different types of CPCs

### Phenotypic differences between Type A and Type B CPC populations

**Figure 3:**
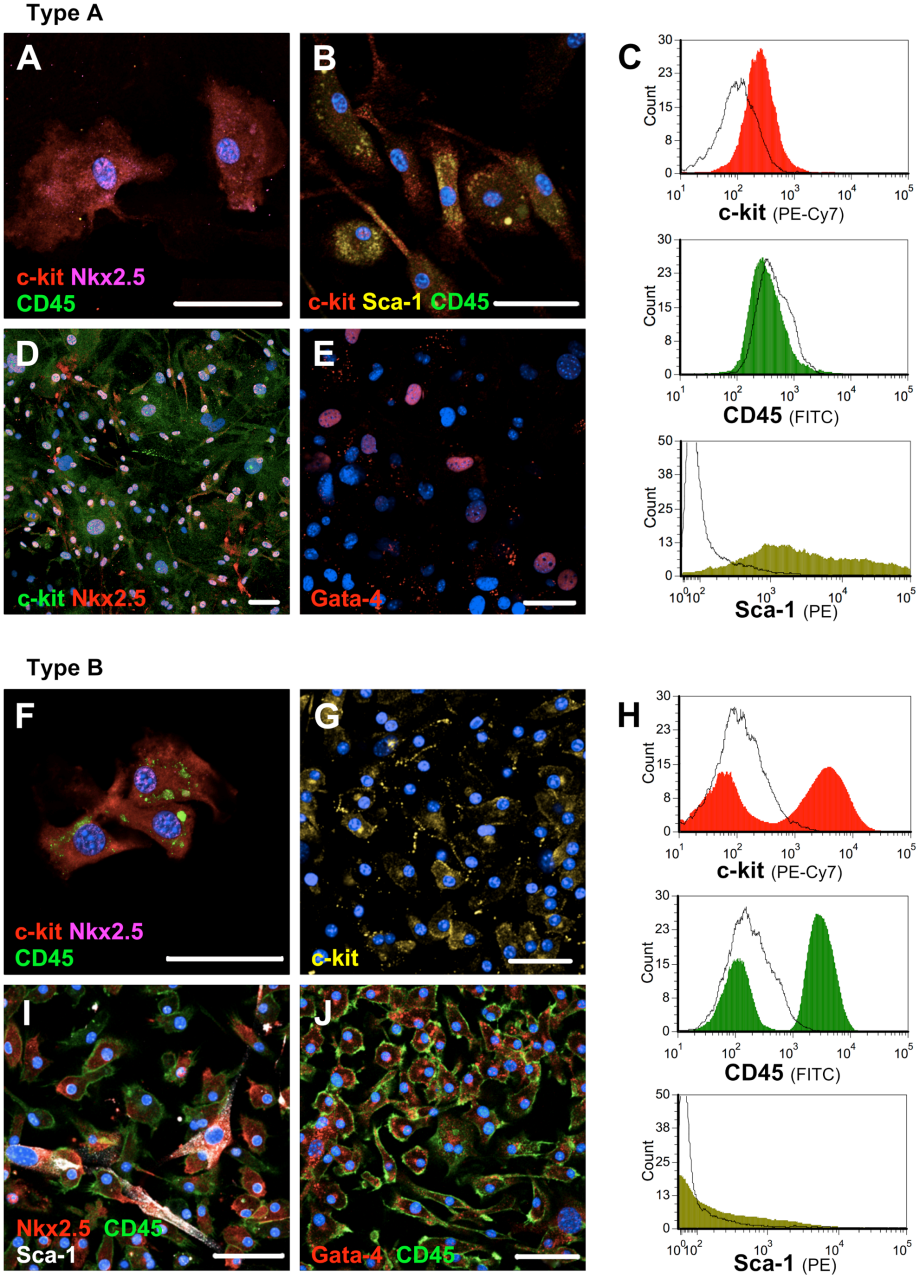
Characterization of Type A and Type B CPC populations. (A and B) C-kit, Sca-1 and Nkx2.5 expression of Type A CPCs after 30 days of culture (1^st^ passage). (C) Flow cytometry results of Type A CPC population after 6 weeks of culture (1^st^ passage). (D and E) c-kit, Nkx2.5 and Gata-4 expression of Type A CPCs after 60 days of culture (2^nd^ passage). (F) c-kit, CD45 and Nkx2.5 expression of Type B CPCs after 30 days of culture (1^st^ passage). (G) c-kit expression of Type B CPCs after 60 days of culture (2^nd^ passage). (H) Flow cytometry results of Type B CPC population after 6 weeks of culture (1^st^ passage). (I and J) CD45, Nkx2.5 and Gata-4 expression of Type B CPCs after 60 days of culture (2^nd^ passage). A minor (∼4%) Sca-1^pos^ and Nkx2.5^pos^ subpopulation is evident. In flow cytometry results: c-kit  =  red, CD45 =  green and Sca-1 =  yellow. Isotype controls of the flow cytometry are shown as black curves. Cells grown from ≧20 LAAs were used in each of the experiments. DAPI  =  blue, bar  = 50 µm. Cells (C and H) were supplemented after the passage with growth factors (1% B27×50, 10 ng/ml EGF, 5 ng/ml bFGF , 10 pmol/L CTF-1, 30 pmol/L Thrombin) for two weeks.

The cell populations had two distinct phenotypes. The slower-growing Type A CPC population expressed surface markers c-kit and Sca-1 (Figure 3A–C), together with Nkx2.5 and Gata-4 (Figure 3D, E; Figure S3A, B, G), which are essential for myocardial development. We were unable to passage the Type A CPC population more than three times, as they reached quiescence. The faster growing Type B CPC population expressed the surface markers c-kit and CD45, but was negative for Sca-1 (Figure 3F-H; Figure S3C, D, G). This population expressed Nkx2.5 and Gata-4 as well (Figure 3I, J). The Type B CPC population was passaged several times and the proliferation rate remained high.

Comparable immunostaining pictures of Type A and Type B CPCs are provided in two different time points: Figures 3 A and B (Type A) and Figure 3F and Figures S3 C and D (Type B) were acquired after 30 days of culture (1st passage). Figures 3 D and E (Type A) and Figures 3 G, I and J (Type B) were acquired after 60 days of culture (2nd passage). Both CPC populations were CD31^neg^ (Figures S3E), ruling out an endothelial progenitor cell -phenotype [22]. Mast Cell-restricted Tryptase (mMCP-6) expression was tested and ruled out mast cell contamination (Figures S3F) [23]. Both cell populations were negative for CD34.

### Cardiomyogenic differentiation of the CPC populations

**Figure 4:**
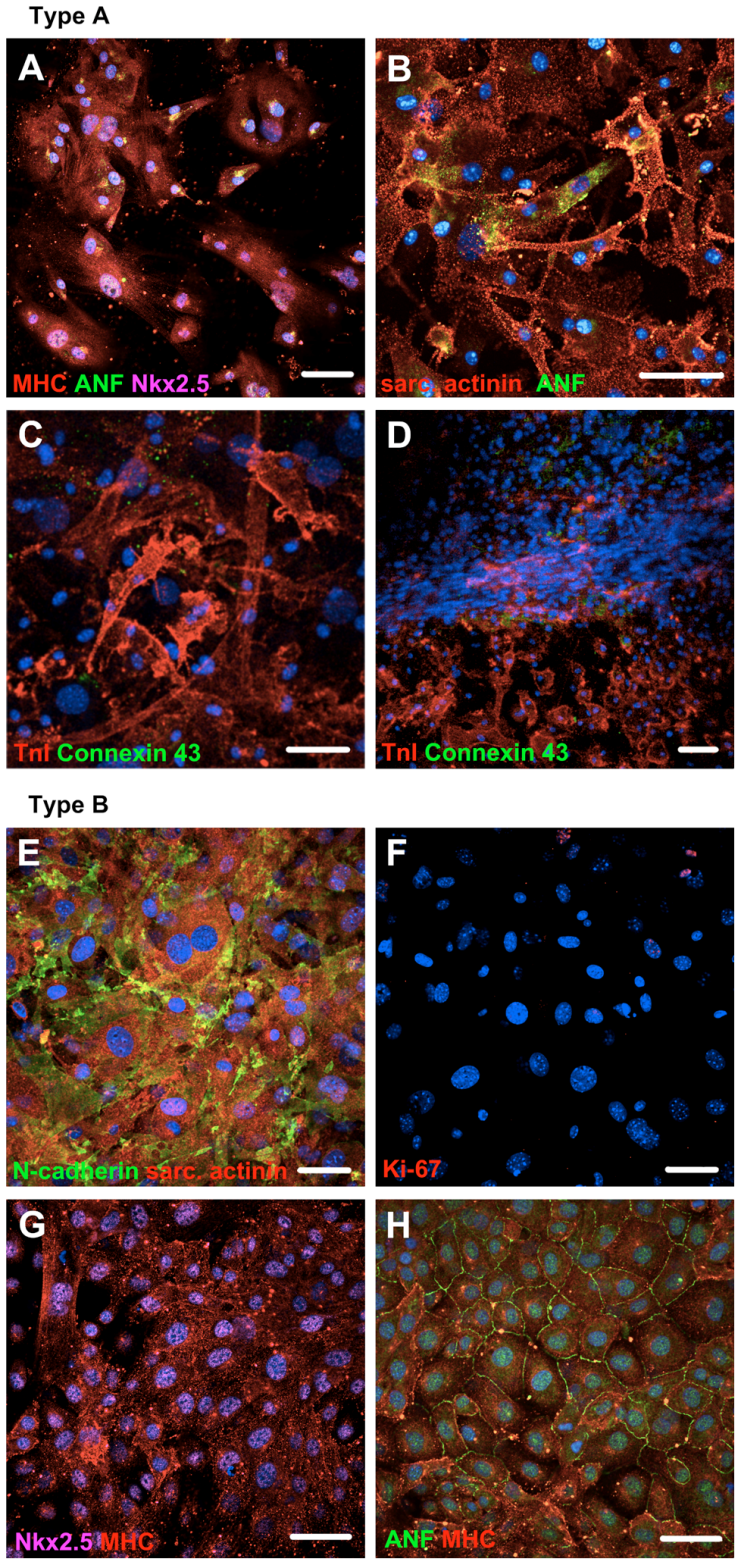
Cardiomyogenic differentiation of Type A and Type B CPC populations. (A and B) Type A CPCs differentiated into cardiomyocytes, when treated with dexamethasone. Organized sarcomeric structures were evident. (C and D) Differentiating cells from Type A CPCs formed multi-layered tissue structure resembling muscle fibers, which expressed TnI and Connexin 43 -gap junctions. (E and F) Type B CPCs differentiated into cardiomyocytes and ceased to proliferate (demonstrated by a lack of Ki-67 staining in majority of cells) when treated with 5 -azacytidine. CD45 expression was confirmed in ∼90% of cells (by FACS) before induction of differentiation. Expression of sarcomeric proteins together with N -cadherin was evident. (G and H) Spontaneous differentiation of Type B CPC population into fast proliferating cells (12^th^ passage). Secreted ANF is seen between the cells. Immunostaining was done three weeks after induction of differentiation. Abbreviations: sarc. actinin  =  Sarcomeric α-actinin, MHC: Cardiac myosin heavy chain; ANF: Atrial natriuretic factor; TnI: Troponin I. DAPI  =  blue, bar  = 50 µm.

The two CPC populations had unique differentiation mechanisms. The Type A CPC population reacted rapidly to dexamethasone: morphology of the cells changed and they gained organized expression of sarcomeric proteins (Figure 4A). The differentiated cells expressed ANF (Figure 4B) and during a three-week follow-up, they formed multi-layered tissue structures resembling muscle fibres. The cells in the fibre and close to it expressed sarcomeric proteins and had Connexin 43 -gap junctions (Figure 4C, D). These fibres reached a length of a few millimetres (Figure S4A–C). Spontaneous contractions were rare, transient and only observed in differentiated Type A CPCs (Movie S1).

The Type B CPC population, on the other hand, after 5 -azacytidine (a DNA demethylation agent) treatment gained an elongated, mature cardiomyocyte-like appearance. The cells expressed sarcomeric proteins and were connected to each other tightly with N-cadherin (Figure 4E). They also acquired senescent state demonstrated by a lack of Ki-67 expression (Figure 4F), one of the main cell adhesion proteins of mature cardiomyocytes. Also, Connexin 43 -gap junctions were present three weeks after 5 -azacytidine treatment (Figure S4D). Type B CPCs had, in addition, a tendency to differentiate spontaneously into more mature cells, while down-regulating CD45 expression. These differentiated cells expressed sarcomeric proteins, but continued to proliferate (Figure 4G,H; Figure S4E, F). Also, ANF was detected in the extracellular space, in addition to nuclear expression. Dexamethasone did not induce a differentiation to Type B CPCs as measured by similar CD45 expression and unchanged cell morphology (data not shown).

### Behavior of Type B CPC population over multiple passages

**Figure 5:**
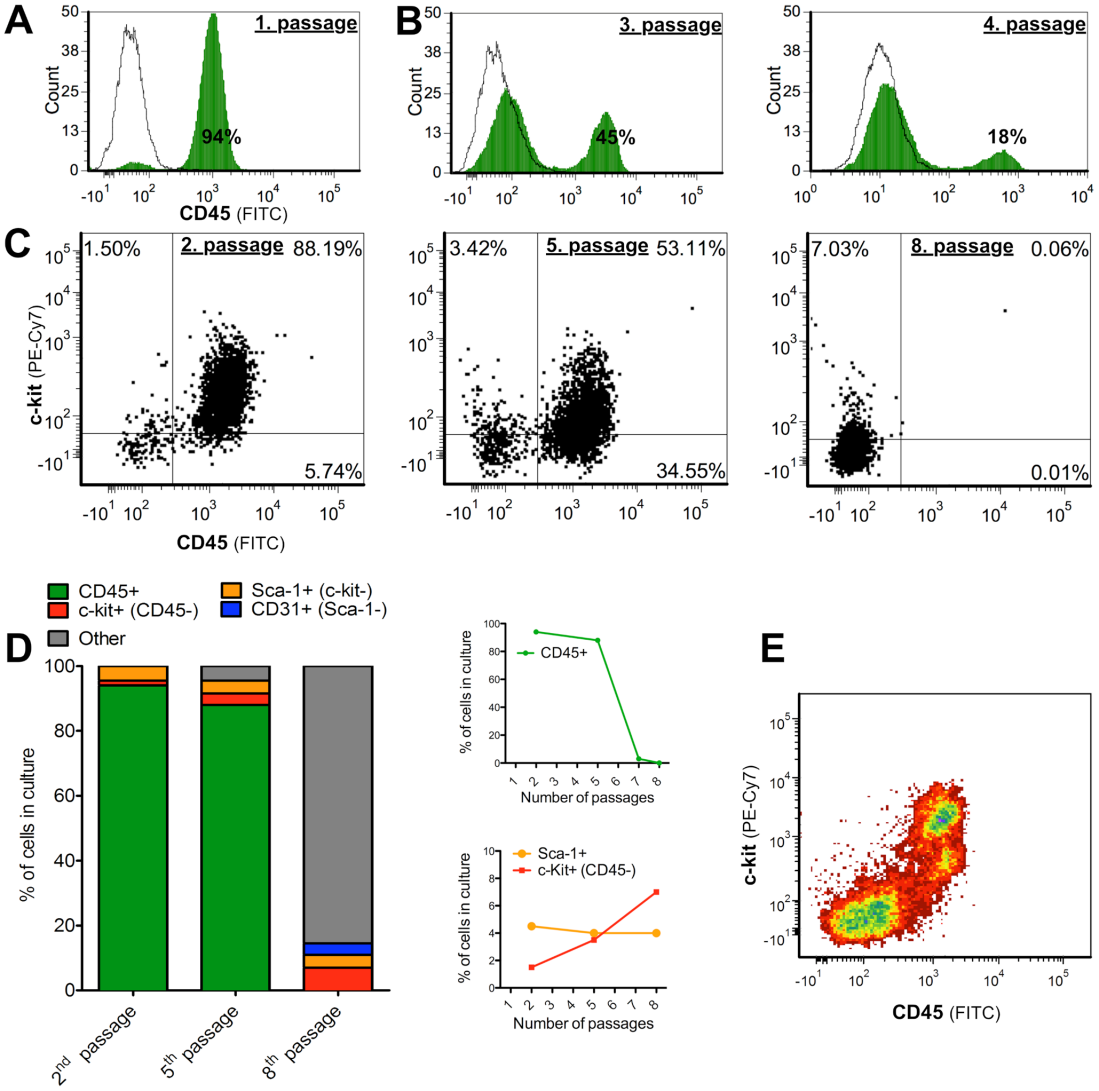
Phenotypic shift of the Type B CPC populations. (A) Our repeated observation was that it was possible to get a ∼95% CD45 and c-kit^pos^ CPC population by doing a passage 14–17 days after establishing the culture. There was no difference found between using a low enzyme concentration or no enzymes at all. (B) A fast down-regulation of CD45 expression between the 3^rd^ and 4^th^ passages. Time between passages was one week. (C) Change of c-kit and CD45 expression in the Type B CPC population over time. Decrease in c-kit expression is observed, followed by a decrease in CD45 expression. (D) Proportions of different subpopulations in the Type B CPC population during different passages. (E) During the spontaneous differentiation c-kit expression is down-regulated first, followed by down-regulation of CD45 expression.

Expression of surface markers is generally used to classify different putative CPC types [24]. We followed the surface marker profile of the Type B CPC population over several passages. Our repeated observation was that it was possible to get a ∼95% CD45 and c-kit^pos^ CPC population by doing the first passage 14–17 days after establishing the culture (Figure 5A). There was no difference found between low enzyme concentration (0.05% trypsin & 0.1% collagenase D) or no enzymes at all. The striking observation was that the proportion of cells expressing CD45 decreased dramatically in a short period of time (Figure 5B). CD45 expression was stable up to five passages, as long as the proportion of CD45^pos^ cells remained ∼90% and the seeding density was held high (∼10^5^ cells/ml) (Figure 5C). Consequently, the decrease in CD45 expressing cells from 45% to 18% happened in only one week (Figure 5B) and the decrease from 88% to 3% happened after two passages (Figure 5C, D; Figure S5A). C-kit expression was down-regulated first and was followed by a down-regulation in CD45 expression (Figure 5E).

We investigated the surface marker profiles of the Type B population cells and found three phenotypically distinct CPC subpopulations: ∼94% CD45^pos^ and Sca-1^neg^ cells, ∼4% Sca-1^pos^ and c-kit^neg^/CD45^neg^ cells and ∼1.5% c-kit^pos^ and Sca-1^neg^/CD45^neg^ cells (Figure 5B, D). The Sca-1^pos^ subpopulation remained at similar level while the c-kit^pos^/CD45^neg^ subpopulation grew slowly during a follow-up of eight passages (Figure 5D; Figure S5B). C-kit and Sca-1 were not expressed in the same cells (Figure S5C). Between the fifth and eight passages, a small subpopulation of cells started to express the epithelial marker CD31 and part of them shared the expression of Sca-1 (Figure S5D).

There was a rapid phenotype shift after the fifth passage. Cells with CD45 expression disappeared, while the proportions of the Sca-1^pos^/c-kit^neg^ and c-kit^pos^/CD45^neg^ CPC subpopulations went through only minor changes. A similar proportion of cells that were CD45^pos^ at the fifth passage did not express any of the surface markers that we investigated (Figure 5D). These cells expressed sarcomeric proteins, together with ANF and Nkx2.5 (Figure 4G, H). All the passages during the follow-up were done in basic culture medium and no differentiation agents were added.

## Discussion

Our results demonstrate that the left atrial appendage plays a role as a reservoir of multiple types of progenitor cells in murine adult hearts. The finding is supported by the early and unique embryonic development of the atrial appendages and their high fetal gene expression [8], [11], which could explain the high number of committed (Nkx2.5^pos^) CPCs found in the LAA [25]. We used three different methods to analyze the amount of c-kit^pos^ cells in the LAA. Tissue analysis by computer software and tissue dissociation showed near equal number of c-kit+ positive cells (∼20-25%), but manual counting suggested the number to be over 30%. In this case, the lower number can be considered to be more accurate, because many more cells were investigated with computer analysis and tissue dissociation.

We were able to grow two distinct CPC populations from the left atrial appendage. Type A CPCs expressed Sca-1 in contrast to Type B CPCs, thus ruling out antigen “peeling” by the enzymatic digestion. The cultured CPCs shared the expression of the progenitor markers found in the tissue, confirming the histological findings. The relationship between growth dynamics and histological location of the Type A (myocardium) and the Type B (epicardium) CPCs was consistent with previous studies [2], [21]. A large number of CPCs, especially CD45 expressing cells, were found in the epicardium, which is a putative region for CPC niches [2]. The Type B population grew only after weak enzymatic digestion, supporting its mainly epicardial localization [6]. These findings support the hypothesis of a layer-specific origin of Type A and Type B CPCs. We cannot rule out the possibility that enzyme digestion compromises the viability of Type B CPCs, and thus preferably promotes growth of Type A CPCs, as we did not perform analysis of the composition of the LAA explant–tissue following different enzyme digestion protocols.

The lack of specific surface markers for identifying CPCs in the adult heart has resulted in the classification of many different cardiac subpopulation cells as potential sources of CPCs [24]. It is unclear whether these subpopulations represent different intermediates originating from common progenitors, or correspond to distinct lineages. A potential explanation could be that the lineage-hierarchy associated with stem cells may be more flexible than is commonly presented [26]. In adult tissues, specialized niche cells supply stem and progenitor cells with paracrine signals necessary for their maintenance or expansion and controls their plasticity. Hypothetically, this control is lost in culture and consequently enables state transitions, which could explain the relatively stable CPC subpopulations seen in the Type B CPC population.

**Figure 6:**
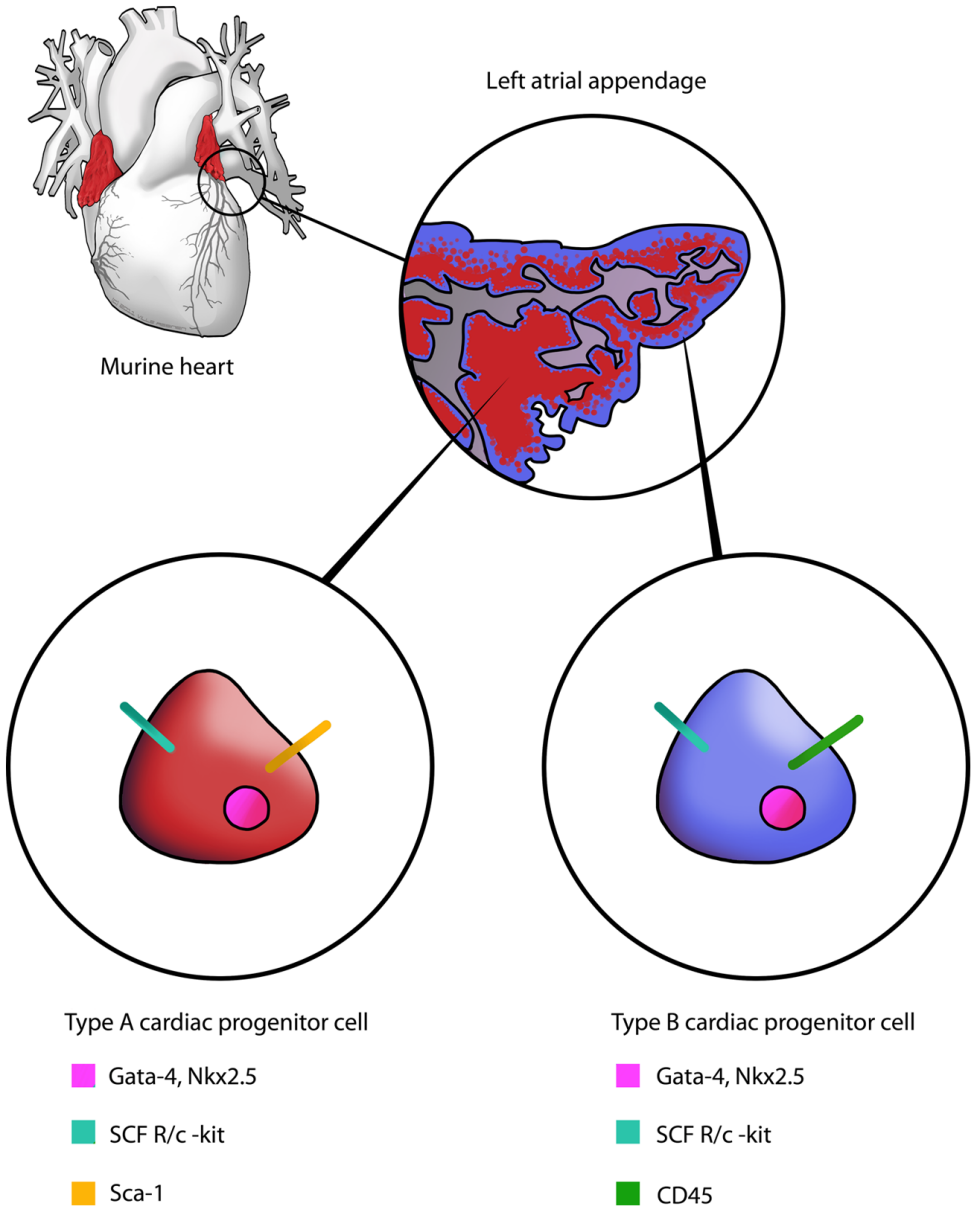
Proposed model of the Type A and B CPC populations residing in the LAA. Type A and Type B CPC populations have a distinct phenotype and preferred histological location in the LAA. Co-residence of the CPC populations suggests that they could contribute to cardiac stem cell niche homeostasis.

In our experiment, the proportions of Sca-1^pos^ and c-kit^pos^ CPC subpopulations underwent only minor changes, while cells co-expressing CD45 and c-kit rapidly decreased in numbers. Simultaneously, a surface-marker negative population, similar in abundance, appeared in the culture. A possible explanation is that the CD45^pos^ CPCs differentiated spontaneously to rapidly- proliferating cells. Other publications support the occurrence of CD45 down-regulation in the c-kit^pos^ cells of the heart [27] and CD45 is shown to be a potent inhibitory factor in differentiation and proliferation through inhibition of the JAK-STAT pathway [28]. Nevertheless, we cannot rule out a cell fusion phenomenon [29], which potentially occurs between CD45^pos^ cells and cardiomyocytes, but this seems unlikely due to the prominent phenotypic shift and lack of multinucleated cells. CD45 has been mostly used as a marker to rule out bone marrow derived inflammatory cells in the field of cardiac regeneration [30]. However; CD45 has a likely role in adult stem cell function. In CD45 deficient (−/−) mice, the most primitive hematopoietic stem cells (HSCs) are reduced in number and CD45 (−/−) bone marrow transplantation recipients show deficient engraftment of normal HSCs, suggesting a role for CD45 in the stem cell niche [31]. Interestingly, HSCs in CD45 (−/−) mice had a deficiency in SDF-1 mediated homing, which is recognized as an important factor in CPC migration [32]. In addition, cardiac cell therapy using bone marrow-derived cells possibly improves cardiac function after myocardial infarction by stimulating resident progenitor cell activity, which raises a question whether Type B CPCs residing in the atrial appendages, like bone marrow cells, may have a role in enhancing the regenerative response [1], [33]. In a majority of publications, CPCs are found to be negative for hematopoietic lineage markers. Nonetheless, some studies have found that a vast amount of c-kit^pos^ CPCs is indeed CD45^pos^ [14], [27]. As our type B CPCs have a spontaneous tendency to rapidly lose CD45 expression, our results could explain this discrepancy. Murine atrial appendages are seldom investigated in cardiac regeneration studies and most of the histological findings come from the ventricles. This is a unique feature of our study, which may explain the large number of CPCs obtained (Figure 6). At present, it is unclear if the progenitor cell populations grown from the left atrial appendage are a distinct pool of progenitors, or represent previously studied progenitors from ventricles of the adult mammalian heart, which possibly migrated from the atrial appendages. The phenotype (c-kit^pos^/lin^neg^) of Type A CPCs and differentiation by dexamethasone induction suggests that they have similar characteristics as extensively studied c-kit^pos^/lin^neg^ cardiac stem cell population [13]. Type B CPCs, on the other hand, share characteristics of c-kit^pos^/CD45^pos^ cells studied by Kubo et al [14]. In future, lineage-tracing experiments will be done to solve this question. We did not establish that the LAA-derived CPCs can achieve full maturation to adult cardiomyocytes. Future cell transplantation experiments will show if the Type A and Type B CPCs can achieve a fully functional adult cardiomyocyte phenotype or contribute to cardiac regeneration by paracrine effects.

Research related to cardiac regeneration in the adult heart is rapidly developing and much of the fundamental information needed is still to be discovered. Future studies will demonstrate if there is a role for the left atrial appendage in homeostasis and regeneration of the adult mammalian heart. This is especially interesting as recently RAA derived c-kit^pos^ CPCs were successfully used in a clinical trial [34]. Our study suggests that epicardial origin may determine different phenotypes and growth dynamics for the CPCs. The reservoir of CPCs in the left atrial appendage raises interesting questions related to a possible migratory mechanism of the CPCs to the rest of the heart. Intriguingly, high density of Nkx2.5^pos^ CPCs in the LAA seems to be located in the epicardial area, which is very much in close contact with the left ventricle. The diversity of progenitor cells in the small anatomical area of the LAA could be used in future studies investigating the function and relationship of different types of CPCs and their milieu.

## Supporting Information

Figure S1 **Nkx2.5, c-kit & CD45 expressing cells in the LAA.**
(TIFF)

Figure S2 **Additional figures of the explant culture.**
(TIFF)

Figure S3 **Additional cell culture analysis.**
(TIFF)

Figure S4 **Differentiation of Type A and Type B CPC populations (additional figures).**
(TIFF)

Figure S5 **Additional flow cytometry results from later passages of Type B CPC population.**
(TIFF)

Movie S1 **Mild spontaneous contractions of a differentiated Type A CPC.**
(MOV)

## References

[1] Loffredo FS, Steinhauser ML, Gannon J, Lee RT (2011) Bone marrow-derived cell therapy stimulates endogenous cardiomyocyte progenitors and promotes cardiac repair. Cell Stem Cell 8: 389–398. 21474103 10.1016/j.stem.2011.02.002 PMC4148018

[2] Smart N, Risebro CA, Melville AA, Moses K, Schwartz RJ, et al (2007) Thymosin beta4 induces adult epicardial progenitor mobilization and neovascularization. Nature 445: 177–182. 17108969 10.1038/nature05383

[3] Itzhaki-Alfia A, Leor J, Raanani E, Sternik L, Spiegelstein D, et al (2009) Patient characteristics and cell source determine the number of isolated human cardiac progenitor cells. Circulation 120: 2559–2566. 19996019 10.1161/CIRCULATIONAHA.109.849588

[4] Vliegen HW, van der Laarse A, Cornelisse CJ, Eulderink F (1991) Myocardial changes in pressure overload-induced left ventricular hypertrophy. A study on tissue composition, polyploidization and multinucleation. Eur Heart J 12: 488–494. 1829680 10.1093/oxfordjournals.eurheartj.a059928

[5] Ptaszek LM, Mansour M, Ruskin JN, Chien KR (2012) Towards regenerative therapy for cardiac disease. Lancet 379: 933–942. 22405796 10.1016/S0140-6736(12)60075-0

[6] Zhou B, Ma Q, Rajagopal S, Wu SM, Domian I, et al (2008) Epicardial progenitors contribute to the cardiomyocyte lineage in the developing heart. Nature 454: 109–113. 18568026 10.1038/nature07060 PMC2574791

[7] Smart N, Bollini S, Dube KN, Vieira JM, Zhou B, et al (2011) De novo cardiomyocytes from within the activated adult heart after injury. Nature 474: 640–644. 21654746 10.1038/nature10188 PMC3696525

[8] Moorman A, Webb S, Brown NA, Lamers W, Anderson RH (2003) Development of the heart: (1) formation of the cardiac chambers and arterial trunks. Heart 89: 806–814. 12807866 10.1136/heart.89.7.806 PMC1767747

[9] Douglas YL, Jongbloed MR, Gittenberger-de Groot AC, Evers D, Dion RA, et al (2006) Histology of vascular myocardial wall of left atrial body after pulmonary venous incorporation. Am J Cardiol 97: 662–670. 16490434 10.1016/j.amjcard.2005.11.019

[10] Al-Saady NM, Obel OA, Camm AJ (1999) Left atrial appendage: structure, function, and role in thromboembolism. Heart 82: 547–554. 10525506 10.1136/hrt.82.5.547 PMC1760793

[11] Houweling AC, van Borren MM, Moorman AF, Christoffels VM (2005) Expression and regulation of the atrial natriuretic factor encoding gene Nppa during development and disease. Cardiovasc Res 67: 583–593. 16002056 10.1016/j.cardiores.2005.06.013

[12] Gambini E, Pompilio G, Biondi A, Alamanni F, Capogrossi MC, et al (2011) C-kit+ cardiac progenitors exhibit mesenchymal markers and preferential cardiovascular commitment. Cardiovasc Res 89: 362–373. 20833650 10.1093/cvr/cvq292

[13] Beltrami AP, Barlucchi L, Torella D, Baker M, Limana F, et al (2003) Adult cardiac stem cells are multipotent and support myocardial regeneration. Cell 114: 763–776. 14505575 10.1016/s0092-8674(03)00687-1

[14] Kubo H, Jaleel N, Kumarapeli A, Berretta RM, Bratinov G, et al (2008) Increased cardiac myocyte progenitors in failing human hearts. Circulation 118: 649–657. 18645055 10.1161/CIRCULATIONAHA.107.761031 PMC2652486

[15] Angert D, Berretta RM, Kubo H, Zhang H, Chen X, et al (2011) Repair of the injured adult heart involves new myocytes potentially derived from resident cardiac stem cells. Circ Res 108: 1226–1237. 21454756 10.1161/CIRCRESAHA.110.239046 PMC3322670

[16] Polesskaya A, Seale P, Rudnicki MA (2003) Wnt signaling induces the myogenic specification of resident CD45+ adult stem cells during muscle regeneration. Cell 113: 841–852. 12837243 10.1016/s0092-8674(03)00437-9

[17] Andersen DC, Andersen P, Schneider M, Jensen HB, Sheikh SP (2009) Murine “cardiospheres” are not a source of stem cells with cardiomyogenic potential. Stem Cells 27: 1571–1581. 19544463 10.1002/stem.72

[18] Pastrana E, Silva-Vargas V, Doetsch F (2011) Eyes wide open: a critical review of sphere-formation as an assay for stem cells. Cell Stem Cell 8: 486–498. 21549325 10.1016/j.stem.2011.04.007 PMC3633588

[19] Urbanek K, Cesselli D, Rota M, Nascimbene A, De Angelis A, et al (2006) Stem cell niches in the adult mouse heart. Proc Natl Acad Sci U S A 103: 9226–9231. 16754876 10.1073/pnas.0600635103 PMC1474010

[20] Wu SM, Fujiwara Y, Cibulsky SM, Clapham DE, Lien CL, et al (2006) Developmental origin of a bipotential myocardial and smooth muscle cell precursor in the mammalian heart. Cell 127: 1137–1150. 17123591 10.1016/j.cell.2006.10.028

[21] Messina E, De Angelis L, Frati G, Morrone S, Chimenti S, et al (2004) Isolation and expansion of adult cardiac stem cells from human and murine heart. Circ Res 95: 911–921. 15472116 10.1161/01.RES.0000147315.71699.51

[22] Sandstedt J, Jonsson M, Lindahl A, Jeppsson A, Asp J (2010) C-kit+ CD45- cells found in the adult human heart represent a population of endothelial progenitor cells. Basic Res Cardiol 105: 545–556. 20119835 10.1007/s00395-010-0088-1

[23] Pouly J, Bruneval P, Mandet C, Proksch S, Peyrard S, et al (2008) Cardiac stem cells in the real world. J Thorac Cardiovasc Surg 135: 673–678. 18329492 10.1016/j.jtcvs.2007.10.024

[24] Sturzu AC, Wu SM (2011) Developmental and regenerative biology of multipotent cardiovascular progenitor cells. Circ Res 108: 353–364. 21293007 10.1161/CIRCRESAHA.110.227066 PMC3073355

[25] Rosenblatt-Velin N, Ogay S, Felley A, Stanford WL, Pedrazzini T (2012) Cardiac dysfunction and impaired compensatory response to pressure overload in mice deficient in stem cell antigen-1. FASEB J 26: 229–239. 21957128 10.1096/fj.11-189605

[26] Gupta PB, Fillmore CM, Jiang G, Shapira SD, Tao K, et al (2011) Stochastic state transitions give rise to phenotypic equilibrium in populations of cancer cells. Cell 146: 633–644. 21854987 10.1016/j.cell.2011.07.026

[27] Fazel S, Cimini M, Chen L, Li S, Angoulvant D, et al (2006) Cardioprotective c-kit+ cells are from the bone marrow and regulate the myocardial balance of angiogenic cytokines. J Clin Invest 116: 1865–1877. 16823487 10.1172/JCI27019 PMC1483161

[28] Irie-Sasaki J, Sasaki T, Matsumoto W, Opavsky A, Cheng M, et al (2001) CD45 is a JAK phosphatase and negatively regulates cytokine receptor signalling. Nature 409: 349–354. 11201744 10.1038/35053086

[29] Alvarez-Dolado M, Pardal R, Garcia-Verdugo JM, Fike JR, Lee HO, et al (2003) Fusion of bone-marrow-derived cells with Purkinje neurons, cardiomyocytes and hepatocytes. Nature 425: 968–973. 14555960 10.1038/nature02069

[30] Chong JJ, Chandrakanthan V, Xaymardan M, Asli NS, Li J, et al (2011) Adult Cardiac-Resident MSC-like Stem Cells with a Proepicardial Origin. Cell Stem Cell 9: 527–540. 22136928 10.1016/j.stem.2011.10.002 PMC3652240

[31] Shivtiel S, Kollet O, Lapid K, Schajnovitz A, Goichberg P, et al (2008) CD45 regulates retention, motility, and numbers of hematopoietic progenitors, and affects osteoclast remodeling of metaphyseal trabecules. J Exp Med 205: 2381–2395. 18779349 10.1084/jem.20080072 PMC2556782

[32] Tang YL, Zhu W, Cheng M, Chen L, Zhang J, et al (2009) Hypoxic preconditioning enhances the benefit of cardiac progenitor cell therapy for treatment of myocardial infarction by inducing CXCR4 expression. Circ Res 104: 1209–1216. 19407239 10.1161/CIRCRESAHA.109.197723 PMC2756190

[33] Hatzistergos KE, Quevedo H, Oskouei BN, Hu Q, Feigenbaum GS, et al (2010) Bone marrow mesenchymal stem cells stimulate cardiac stem cell proliferation and differentiation. Circ Res 107: 913–922. 20671238 10.1161/CIRCRESAHA.110.222703 PMC3408082

[34] Bolli R, Chugh AR, D'Amario D, Loughran JH, Stoddard MF, et al (2011) Cardiac stem cells in patients with ischaemic cardiomyopathy (SCIPIO): initial results of a randomised phase 1 trial. Lancet 378: 1847–1857. 22088800 10.1016/S0140-6736(11)61590-0 PMC3614010

