# Supplementary figures and images for: Left Atrial Appendages from Adult Hearts Contain a Reservoir of Diverse Cardiac Progenitor Cells
Source: PLoS One. 2013 Mar 12;8(3):e59228. doi: 10.1371/journal.pone.0059228 (PMC3595246; doi:10.1371/journal.pone.0059228)

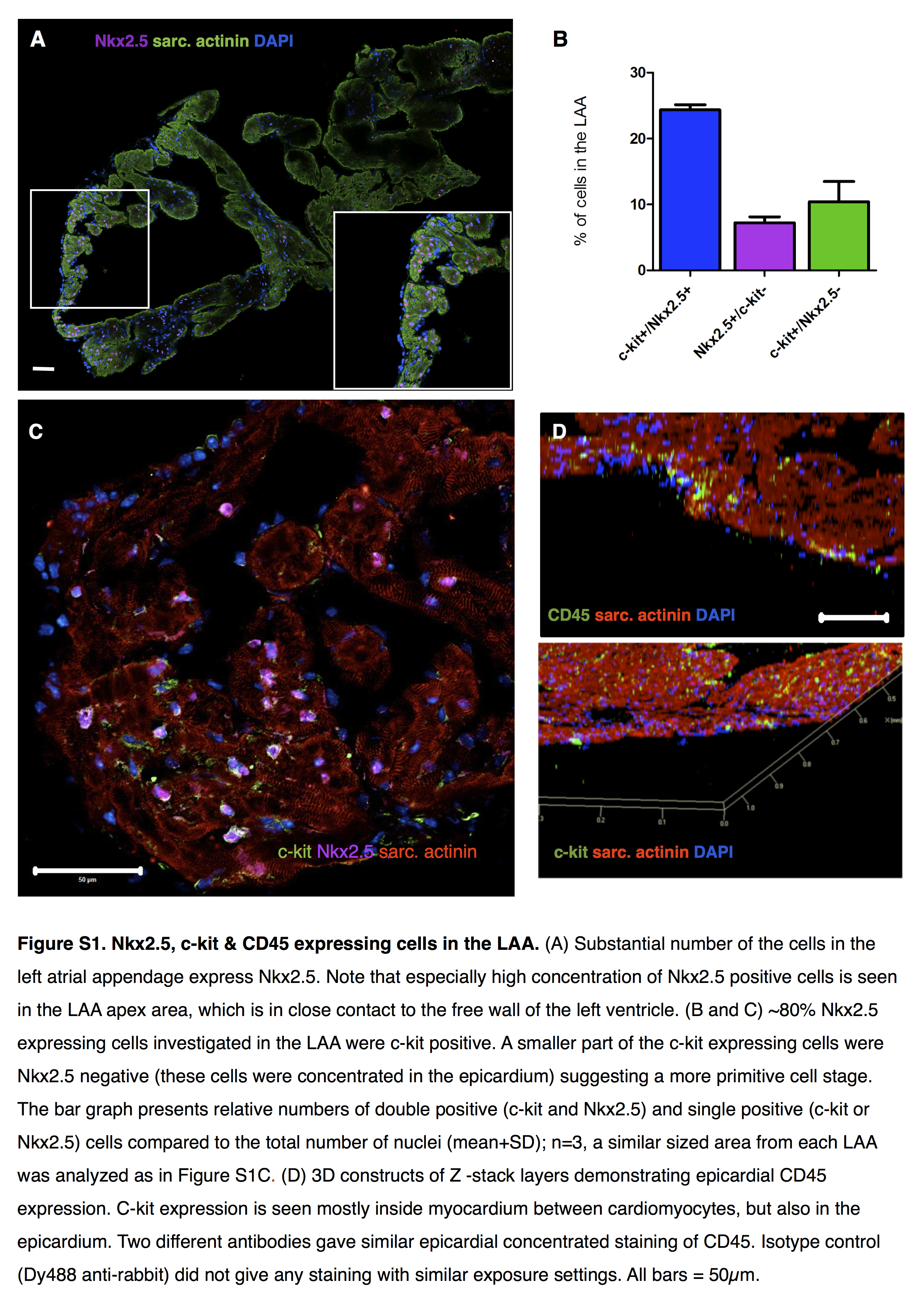

Supplement: Figure S1 — Nkx2.5, c-kit & CD45 expressing cells in the LAA. (TIFF) [file pone.0059228.s001.tif]

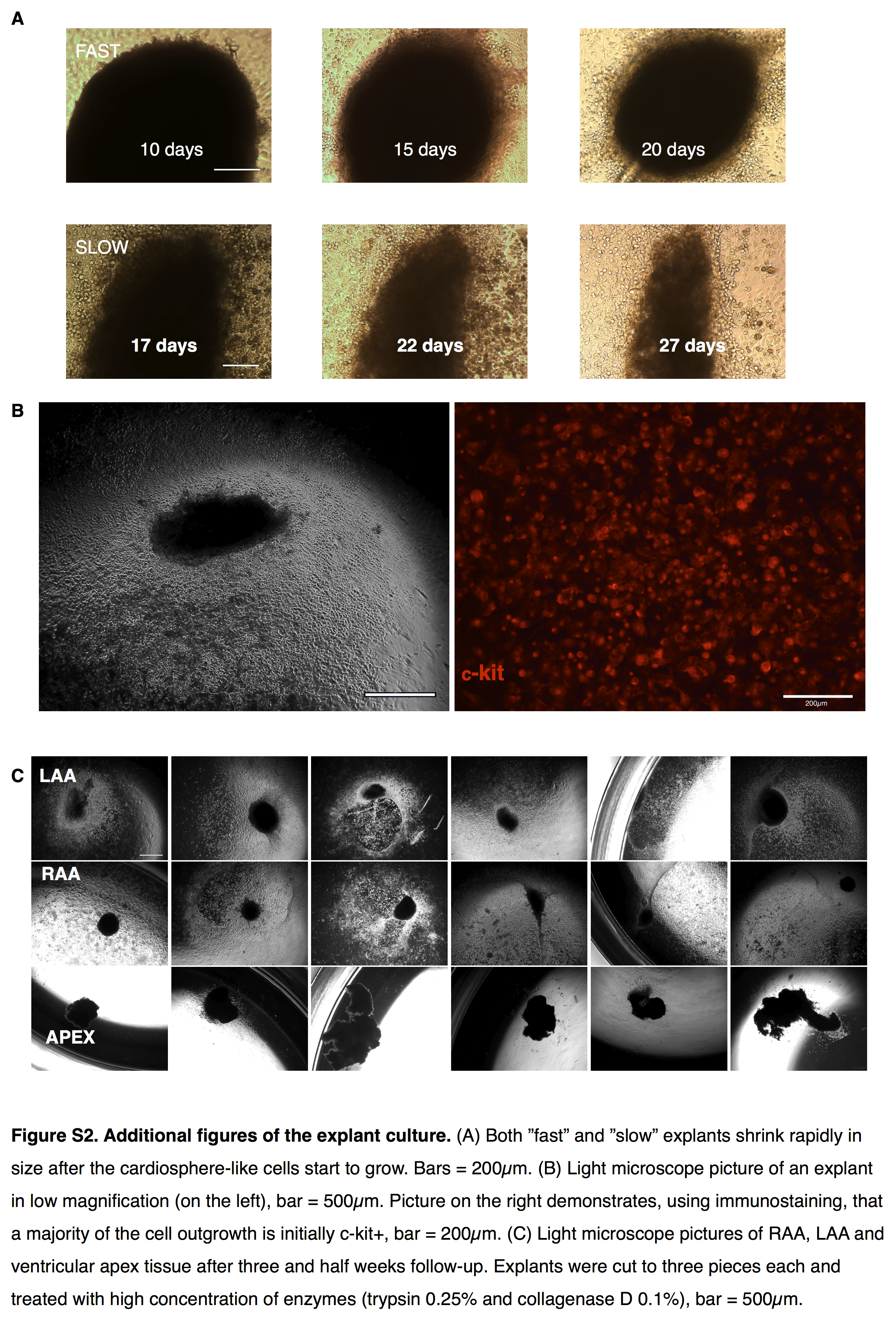

Supplement: Figure S2 — Additional figures of the explant culture. (TIFF) [file pone.0059228.s002.tif]

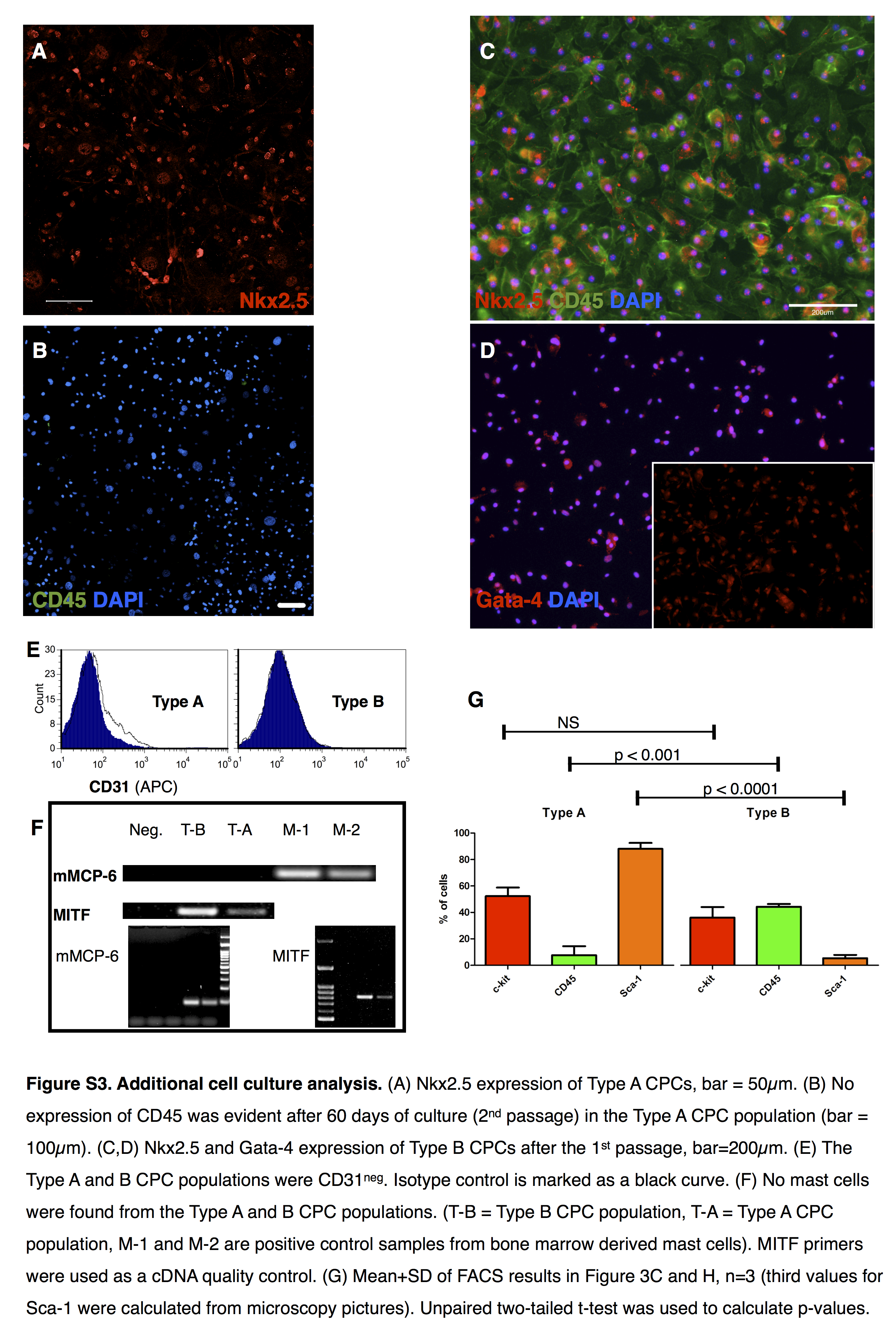

Supplement: Figure S3 — Additional cell culture analysis. (TIFF) [file pone.0059228.s003.tif]

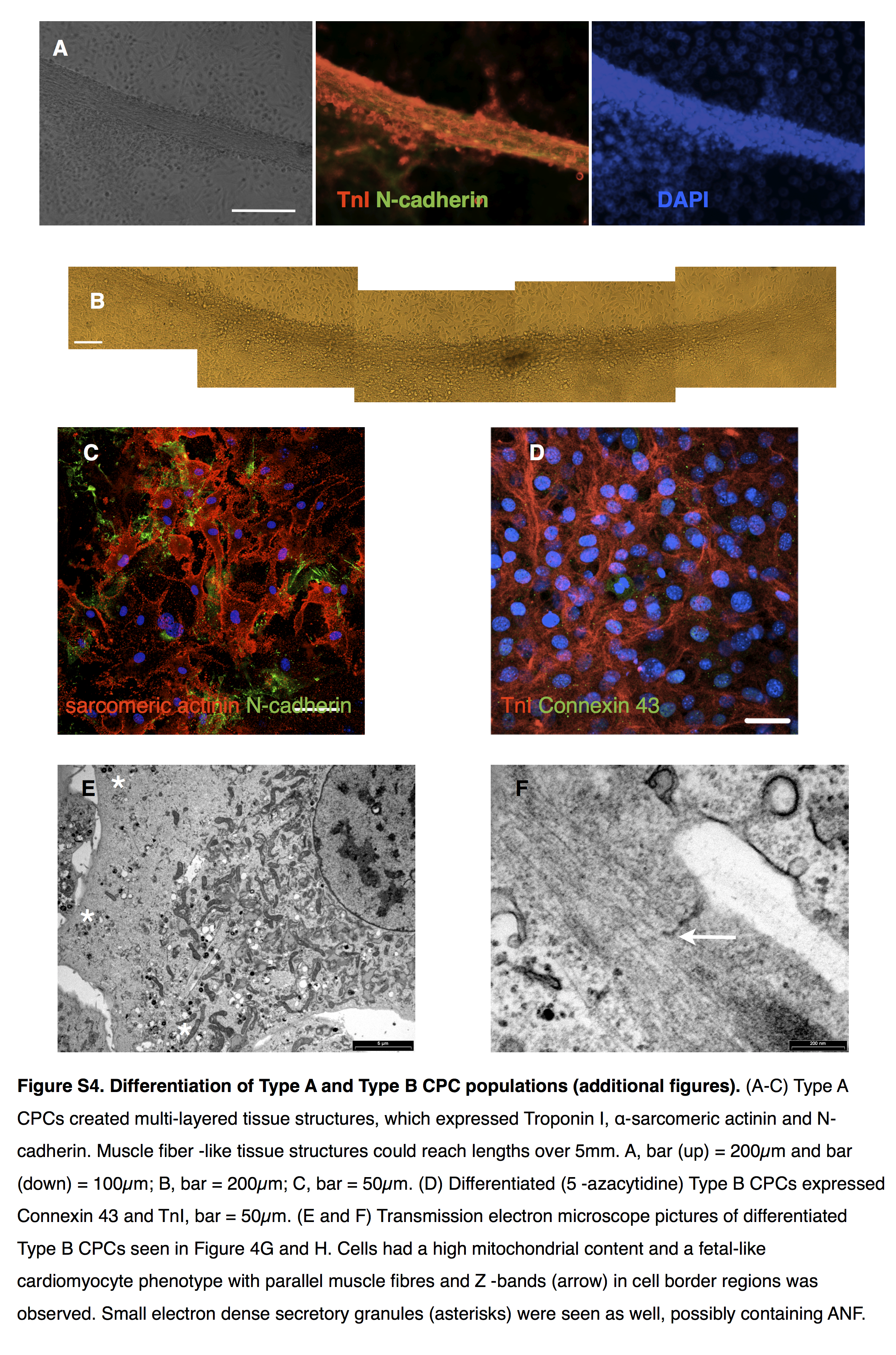

Supplement: Figure S4 — Differentiation of Type A and Type B CPC populations (additional figures). (TIFF) [file pone.0059228.s004.tif]

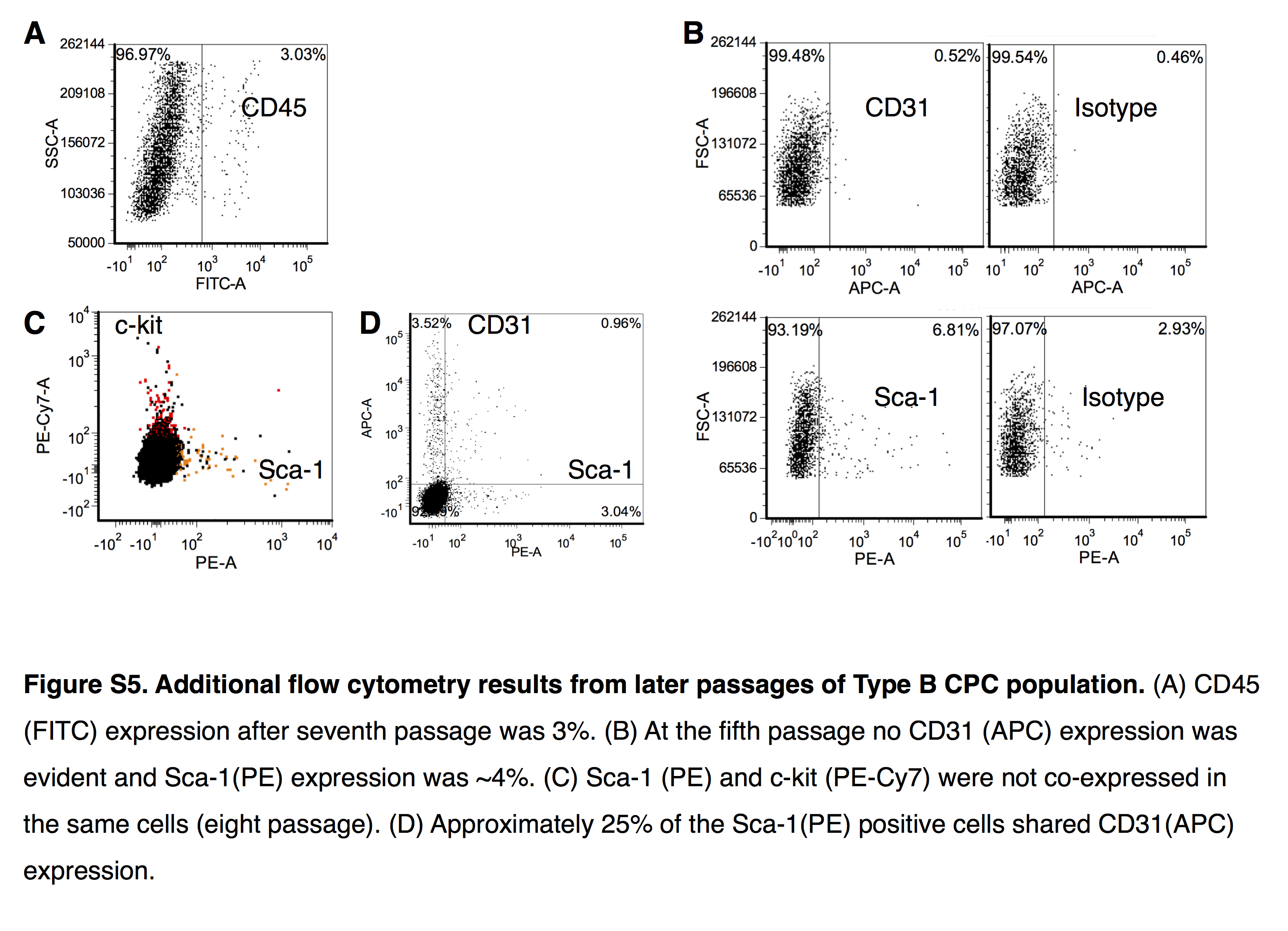

Supplement: Figure S5 — Additional flow cytometry results from later passages of Type B CPC population. (TIFF) [file pone.0059228.s005.tif]
